# Structural homeostasis in the nervous system: a balancing act for wiring plasticity and stability

**DOI:** 10.3389/fncel.2014.00439

**Published:** 2015-01-20

**Authors:** Jun Yin, Quan Yuan

**Affiliations:** National Institute of Neurological Disorders and Stroke, National Institutes of HealthBethesda, MD, USA

**Keywords:** structural plasticity, homeostasis, neural development, neuronal morphology, activity-dependent plasticity

## Abstract

Experience-dependent modifications of neural circuits provide the cellular basis for functional adaptation and learning, while presenting significant challenges to the stability of neural networks. The nervous system copes with these perturbations through a variety of compensatory mechanisms with distinct spatial and temporal profiles. Mounting evidence suggests that structural plasticity, through modifications of the number and structure of synapses, or changes in local and long-range connectivity, might contribute to the stabilization of network activity and serve as an important component of the homeostatic regulation of the nervous system. Conceptually similar to the homeostatic regulation of synaptic strength and efficacy, homeostatic structural plasticity has a profound and lasting impact on the intrinsic excitability of the neuron and circuit properties, yet remains largely unexplored. In this review, we examine recent reports describing structural modifications associated with functional compensation in both developing and adult nervous systems, and discuss the potential role for structural homeostasis in maintaining network stability and its implications in physiological and pathological conditions of the nervous systems.

## Introduction

The structural organization of the nervous system has been studied since the earliest days of neuroscience. Before the wide use of electrophysiology, anatomical study was the main approach for neuroscientists to investigate the organization of the nervous system and infer principles governing its operation. Since then, samples from various species and developmental stages revealed remarkable complexity, diversity, and flexibility in neuronal forms and connections, which are mainly determined by each individual’s genetic composition, but also largely influenced by experience and environmental factors (Holtmaat and Svoboda, 2009; Fu and Zuo, 2011). Although most prominent during development, structural plasticity is also evident in adult brains, serving critical cognitive functions such as learning and memory (Goodman and Shatz, 1993; Katz and Shatz, 1996; Chklovskii et al., 2004; Lamprecht and LeDoux, 2004).

As a fundamental property of the nervous system, its functional and structural flexibility provides the ability to adapt and incorporate genetic, developmental, and environmental variations, but at the same time, poses significant challenges to the integrity of neural networks. Therefore, counterbalancing mechanisms that maintain network stability are critically important. Observations in the central and peripheral nervous systems of various model organisms validated the existence of compensatory regulatory mechanisms, which are defined as neuronal homeostasis (Turrigiano and Nelson, 2000; Davis, 2013). In contrast to the classic Hebbian form of plasticity, where positive feedback regulation reinforces activity-induced changes and leads to long-lasting synaptic plasticity (Turrigiano and Nelson, 2000; Malenka and Bear, 2004; Cooper and Bear, 2012), homeostatic plasticity constrains network activity within the target physiological limit in response to changes of synaptic or intrinsic activity (Davis and Bezprozvanny, 2001; Turrigiano and Nelson, 2004; Marder and Goaillard, 2006; Turrigiano, 2012). Hebbian and homeostatic plasticity are opposing forces that potentially drive neuronal changes in different directions. Recent findings revealed both convergent and distinct molecular pathways underlying these two forms of plasticity. Mechanisms regulating their intricate interplay are clearly important for the nervous system to achieve proper balance between flexibility and stability, but remain largely unknown (Vitureira and Goda, 2013).

Based on the current understanding of underlying cellular mechanisms, neuronal homeostasis is generally categorized as the homeostatic control of: (a) intrinsic excitability through the regulation of ion channel expression; (b) synaptic efficacy through the synaptic scaling at the postsynaptic density (PSD); (c) release of presynaptic neurotransmitter; and (d) network activity through regulation of inhibitory synapses (Turrigiano and Nelson, 2000, 2004; Davis, 2013). Most of the studies related to neuronal homeostasis focused on modulation occurring at the level of synaptic physiology. However, under conditions where synaptic homeostasis is induced, concomitant morphological changes were also observed (Murthy et al., 2001; Butz et al., 2009b; Keck et al., 2013). More importantly, it is clear that some of the activity-dependent structural plasticity did not follow the Hebbian learning rules, since they act on a global scale rather than being restricted within specific synapses, and produce counterbalancing rather than reinforcing effects on activity-driven changes, suggesting a structural realization of the homeostatic regulation (Tripodi et al., 2008; Butz et al., 2009b; Yuan et al., 2011; Keck et al., 2013). Are these structural modifications merely adaptive responses reacting to the alterations in synaptic physiology, or a part of the homeostatic mechanism that is actively contributing to the stabilization of the network activity? Answering this question is potentially important for us to gain a comprehensive understanding on how neural plasticity is cooperatively regulated through distinct mechanisms, and how the balance between wiring dynamics and network stability are achieved and modified during development and in adulthood. Here, we summarize observations generated from a diverse group of model systems that suggest a close association between structural plasticity and neuronal homeostasis, and discuss how these studies might support structural homeostasis as a conserved mechanism in regulating neuronal function, as well as its implications in physiological and pathological conditions.

## Structural plasticity associated with homeostasis at the excitatory synapse

**Figure 1:**
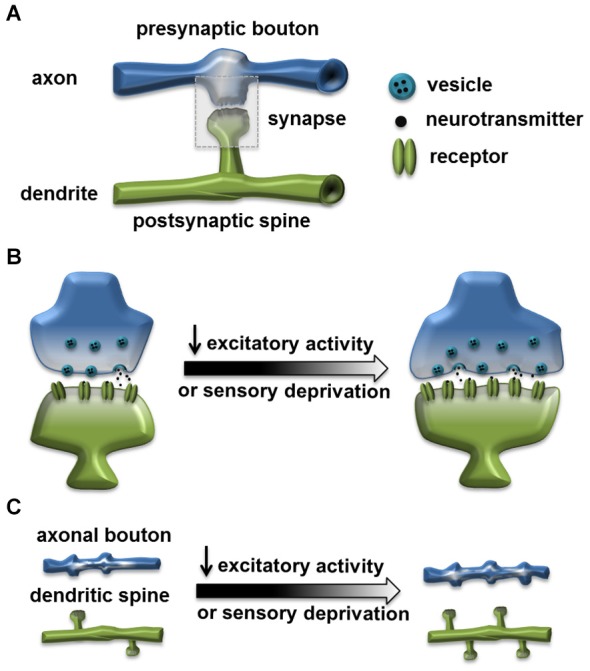
**Homeostatic structural change at the excitatory synapse. (A)** A typical excitatory synapse. Synapse is formed through connection of the presynaptic axonal bouton, containing the presynaptic active zone (AZ) with the readily releasable neurotransmitter vesicles, and the postsynaptic dendritic spine, containing the postsynaptic density (PSD) that organizes neurotransmitter receptor clusters. **(B)** Reduced excitatory activity or sensory deprivation can lead to structural changes including: enlarged spine, increased size of active zone and increased number of vesicles (Murthy et al., 2001; Wallace and Bear, 2004; Keck et al., 2013). **(C)** Reduced excitatory activity or sensory deprivation can also lead to an increased axonal bouton number (Yamahachi et al., 2009; Kremer et al., 2010); increased dendritic spine number (Kirov and Harris, 1999); or reduced spine elimination rate (Zuo et al., 2005).

Activity-dependent structural plasticity at the excitatory synapse has been an intensely investigated research topic for decades. Structural modifications accompanying experience, such as spine growth induced by learning, are proposed to be the cellular mechanism underlying cognitive functions and behavioral plasticity (Chklovskii et al., 2004; Lamprecht and LeDoux, 2004; Holtmaat and Svoboda, 2009). In mammalian nervous systems, the organization and composition of excitatory synapses are well characterized. They generally form between the presynaptic bouton, containing the presynaptic active zone (AZ) with the readily releasable neurotransmitter vesicles, and the postsynaptic spine, containing the PSD that organizes neurotransmitter receptor clusters. In general, the size of a synapse or synaptic components is positively correlated with the synaptic efficacy and strength. Neuronal activity can strongly influence the size and structure of a synapse, as well as its distribution and dynamics (Figure 1; Murthy et al., 2001; Holtmaat and Svoboda, 2009).

Many classic studies on neuronal homeostasis focused on alterations in synaptic strength and efficacy upon chronic changes in neuronal activity, either through pharmacological treatments that artificially increase or decrease neural activity, or sensory deprivation (Turrigiano and Nelson, 2000; Turrigiano, 2008; Pozo and Goda, 2010; Cooper and Bear, 2012), while structural modifications were also observed under similar induction protocols. For example, early morphological studies in hippocampal slices from adult rat showed that dendrites became more spiny in slices with blocked synaptic transmission (Kirov and Harris, 1999). In a series of experiments that established homeostasis as a distinct form of synaptic plasticity, chronic blockade of cortical culture activity with the sodium channel blocker tetrodotoxin (TTX) not only enhanced synaptic strength through synaptic scaling at the PSD (Turrigiano et al., 1998), but also led to increased synaptic size, with all synaptic components, including the AZ, PSD and the bouton becoming larger (Murthy et al., 2001). In addition, to improve spatial and temporal resolutions to manipulate neuronal activity, researchers used ectopic expression of potassium channel Kir2.1 to suppress excitability in cultured hippocampal neurons (Burrone et al., 2002). Chronic suppression of activity after synapse formation in individual neurons within an active network led to homeostatic increase in synaptic input strength, and the total recycling pool of vesicles enlarged in synapses terminating on Kir2.1-expressing neurons (Burrone et al., 2002). Importantly, this study also indicated that timing of the activity modification and competitions among synapses strongly influence expression of synaptic homeostasis.

Taking advantage of the advancement in imaging techniques, *in vivo* experiments in mammalian sensory systems provided additional evidence for the structural changes associated with neuronal homeostasis. In the somatosensory system, long-term sensory deprivation in mice through whisker trimming results in a reduced rate of ongoing spine elimination in the barrel cortex. Since there are continuous synapse and spine loss after birth, the reduced spine elimination might be a way to compensate for the loss of sensory inputs (Zuo et al., 2005). More recently, studies were carried out in the mouse visual cortex with sensory deprivation generated through precise focal retinal lesions. The synaptic activity in the cortical neurons was initially decreased significantly, but gradually recovered within 2 days. This was coincident with enlarged spine size in layer 5 pyramidal neurons in the projection zone (Keck et al., 2013).

As an important mechanism for neuronal homeostasis, homeostatic regulation of presynaptic neurotransmitter release was demonstrated in the* Drosophila* neuromuscular junction (NMJ; Davis and Bezprozvanny, 2001; Frank, 2014). Depleting postsynaptic glutamate receptor subunits GluRIIA or GluRIIC reduced quantal size and elevated presynaptic release (Petersen et al., 1997; DiAntonio et al., 1999; Marrus et al., 2004). Moreover, a number of genetic mutations associated with diminished glutamate receptor clusters at NMJ also show reduced quantal size coupled with a compensatory increase in quantal content (Albin and Davis, 2004; Heckscher et al., 2007). Although the homeostatic regulation of synaptic physiology is very robust in NMJ, clear compensatory structural modifications were not observed in this system, suggesting that expression of synaptic homeostasis may be strongly influenced by induction methods and neuronal types.

Taken together, there is evidence suggesting that structural modifications of synaptic compartments are associated with the neuronal homeostasis. However, the results are mixed and it is clear that functional synaptic homeostasis can exist alone without obvious morphological alterations. Current limited data suggest that structural homeostasis may share molecular mechanisms with functional synaptic homeostasis. In several cases, structural homeostasis is correlated with “synaptic scaling” which involves trafficking of postsynaptic AMPA and NMDA receptors. In visually deprived animals, the number of AMPA receptors in the spine increased in parallel with the enlarged neuron spine head (Wallace and Bear, 2004; Keck et al., 2013). Mice with blocked NMDA receptors showed decreased spine elimination rate in the brain, similar to the homeostatic change in dynamics caused by whisker trimming (Zuo et al., 2005). But, in general, the information on molecular signaling pathways for structural synaptic homeostasis remains largely unknown. It is likely that physiological and structural changes collaborate in the process of re-establishing network stability, but occur within different temporal and spatial scales, and might be induced through both shared and distinct mechanisms.

## Large-scale structural modifications associated with neuronal homeostasis

Although some homeostatic regulations act in a synapse-specific manner, in general, neuronal homeostasis involves organized responses within a neuron or a neural network composed of many synaptic connections (Turrigiano, 2012). Therefore, to better understand the relation between structural and functional homeostatic plasticity, it is essential to analyze the overall change of network structure with correlative physiology data. This is challenging because of the extensive neuronal projections and synaptic connections made by mammalian cortical neurons. Nonetheless, several groups demonstrated large-scale reorganizations of axonal or dendritic compartments associated with homeostasis in both vertebrate and invertebrate systems.

### Axonal sprouting associated with reduced visual activity

Activity-dependent reorganization at the level of axonal and dendritic arbors is traditionally associated with the developing nervous system (Antonini and Stryker, 1993; Portera-Cailliau et al., 2005). In an adult brain, neuronal connections remain relatively stable and most of the structural modifications are observed at the synaptic level. Yet, large scale structural changes, such as axonal or dendritic arbor dynamics were demonstrated in adult brains, providing the cellular basis for the sustained structural flexibility in fully developed neural circuits (Fu and Zuo, 2011). For example, De Paola et al. (2006) reported prominent plasticity of axonal arbors in mouse barrel cortex through long-term *in vivo* imaging and demonstrated important rewiring ability in the axonal compartment of mature neurons.

Specifically, *in vivo* imaging studies in the primate visual cortex showed compensatory axonal sprouting associated with reduced visual activity. In animals with retinal lesions, an increased number and turnover rate of axonal boutons in the lesion projection zone (LPZ) were observed (Yamahachi et al., 2009). Moreover, focal binocular retinal lesions generated large-scale axonal sprouting and pruning in long-range horizontal axons within the LPZ, followed by proliferation of the horizontal axons at a high rate with density peaking within 1 week. Although the axon elimination rate also increased in the later stage, the overall axon density remained elevated during the whole observation period of 7–8 weeks, suggesting a large-scale and long-lasting reorganization of axonal projections in response to reduced visual input (Yamahachi et al., 2009).

### Homeostatic structural tuning of the axon-initial segment

The axon-initial-segment (AIS) is a highly specialized structure that separates axonal and somato-dendritic compartments. Axon-initial-segment maintains neuronal polarity by filtering the cellular cargo, functions as the trigger zone for action potentials, and is involved in modulating complex neuronal processing (Grubb et al., 2011). Shifting the AIS location and changing its size potentially affect the speed of electric signal propagation and the intrinsic excitability of the neuron. With its unique molecular composition, AIS provides an opportunity for morphological and functional studies following activity modification (Grubb and Burrone, 2010; Gründemann and Häusser, 2010; Kuba et al., 2010; Kuba, 2012).

**Figure 2:**
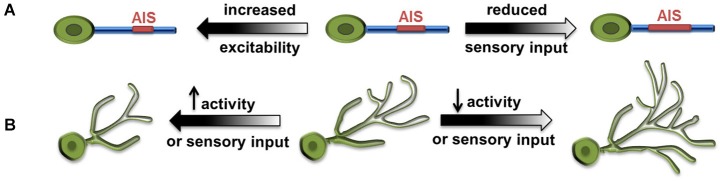
**Large-scale homeostatic structural changes. (A)** Increased excitability results in distal shift of axonal initial segment (AIS) from the soma and reduced AIS length (Grubb and Burrone, 2010), whereas reduced sensory input results in increased AIS length and reduced distance to soma (Kuba et al., 2010). **(B)** Increased synaptic activity and excitability, as well as increased sensory input lead to decreased size of dendritic arbors. Conversely, reduced activity or visual input leads to increased size of dendritic arbors (Tripodi et al., 2008; Yuan et al., 2011).

Two research groups examined activity-dependent structural dynamics of the AIS recently and convincingly demonstrated homeostatic structural tuning in vertebrate neurons. In cultured hippocampal neurons, chronically elevated intrinsic activity through the action of channel rhodopsin (ChR2), or increased extracellular potassium, resulted in a distal shift of AIS from the soma and a reduction in AIS length (Grubb and Burrone, 2010). Along with this distal shift, there was a relocation of AIS-specific proteins including several ion channel components. This distal movement of ion channels reduced the ability of the input to trigger action potentials. Thus, the movement of the AIS away from the soma reduced excitability to compensate for increased neuronal activity. Notably, the AIS relocation through elevated activity was reversible, as AIS shifted proximally toward the soma after the activity returned to baseline, suggesting a regulatory mechanism for fine-tuning the AIS structure base on the ongoing activity (Figure 2A; Grubb and Burrone, 2010).

Similar observations were made in neurons in the nucleus magnocellularis (NM) of the chick auditory system, where sound frequency tuned the structural properties of AIS bi-directionally. High-frequency sounds produced less auditory input and led to shorter AIS, and vice versa (Kuba and Ohmori, 2009). In a follow-up study, Kuba et al. (2010) further showed that, when auditory activity was abolished by removing the cochlea in chickens, the AIS in deprived NM neurons elongated more than 50% and its distance to the soma decreased as well.

Using complementary approaches, these two studies clearly demonstrated homeostatic modification of the length and location of AIS. These observations strongly suggest that homeostatic structural plasticity of AIS can effectively modulate neuronal function and serve as a mechanism for tuning neuronal activity based on sensory input.

### Homeostatic modification of the dendritic arbors in *Drosophila* central neurons

*Drosophila* neurobiology has contributed significantly to our knowledge of the principles governing neural development and circuit organization. Recent studies demonstrated structural modifications associated with neuronal homeostasis in several types of *Drosophila* central neurons. The first example of homeostatic remodeling of dendritic arbors came from the study on aCC, a group of embryonic motor neurons located in the ventral nerve cord and receiving cholinergic inputs (Tripodi et al., 2008). Blocking the neurotransmitter synthesis or evoked release from presynaptic cholinergic neurons both led to expansion of the aCC dendritic arbor. Conversely, when the density of presynaptic contacts and synapses formed on dendrites was increased by genetic manipulation, the size of the aCC dendritic arbor was significantly reduced (Figure 2B). The authors proposed that the dendrite of aCC motor neurons exhibits activity-dependent structural homeostasis, which could serve as a compensatory mechanism for neurons to cope with the variation of presynaptic inputs throughout development (Tripodi et al., 2008).

The mushroom body is a well-studied structure in the *Drosophila* central nervous system (CNS) due to its close association with learning and memory, sensory integration and behavioral plasticity. Early studies showed that the volume of mushroom body can be modified by culture conditions and sensory experience (Heisenberg et al., 1995). Recently, Kremer et al. (2010) studied experience-dependent modification of synaptic structures in the adult mushroom body through high-resolution imaging. What they have found was unexpected for the structure associated with memory, where lack of input was assumed to lead to reduced complexity and volume. Instead, when input activity was silenced through ectopic expression of a potassium channel dORK1.ΔC in presynaptic projection neurons, they observed a significant increase in the density and size of the microglomerulus, the synaptic complex formed between axon terminals from projection neurons and postsynaptic structure of Kenyon cells in the mushroom body. This suggests a possible homeostatic upregulation of the microglomerulus synapse in response to suppression of neuronal activity (Kremer et al., 2010). However, the activity manipulation was carried out only in presynaptic neurons and it was not restricted to adulthood. With improved temporal control and additional studies in postsynaptic mushroom body neurons, future work using this system might provide us with more information regarding the extent and location of these homeostatic structural changes, as well as how this type of regulation interacts with the sensory integration and learning activity in the mushroom body.

Our study using the developing larval visual system further demonstrated structural homeostasis in *Drosophila* CNS and depicted novel features and molecules involved in its regulation (Yuan et al., 2011). In *Drosophila* larvae, presynaptic photoreceptors send an axonal projection to the brain and make synaptic contacts with dendritic arbors of ventral lateral neurons (LNv). Light stimulation-induced synaptic activity produced striking changes in the length of LNv dendrites, with the amount of light exposure received by the animal inversely correlated with the total dendritic length of LNv (Yuan et al., 2011). Through neuronal-specific alterations of activity and neurotransmission, we demonstrated compensatory structural changes in dendritic arbors driven by either sensory experience, or alterations in the presynaptic neurotransmission or synaptic activity, or changes in the intrinsic excitability in postsynaptic neurons (Figure 2B). This large-scale, bi-directional and homeostatic structural plasticity is accompanied by changes in the LNvs’ physiological output. Light-evoked activity in LNvs, measured by calcium imaging, are also modified by experience, where increased light exposure correlated to reduced dendrite length and lower physiological response in LNvs. This observation clearly contrasts with the classic homeostasis theory, in which a set point that precisely defines the network output is a major feature (Davis, 2006).

In combination, these three studies demonstrated structural homeostasis associated with distinct developmental stages: embryonic motor neurons undergoing active synaptogenesis, larval LNv extending its connectivity with the expansion of the brain volume, and adult mushroom body neurons responding to the learning or experienced-dependent modification. These results support the idea that *Drosophila* central neurons can continually utilize structural modifications as a mechanism for modifying functional output according to developmental and environmental influences.

## Structural plasticity associated with neuronal homeostasis at the inhibitory synapse

**Figure 3:**
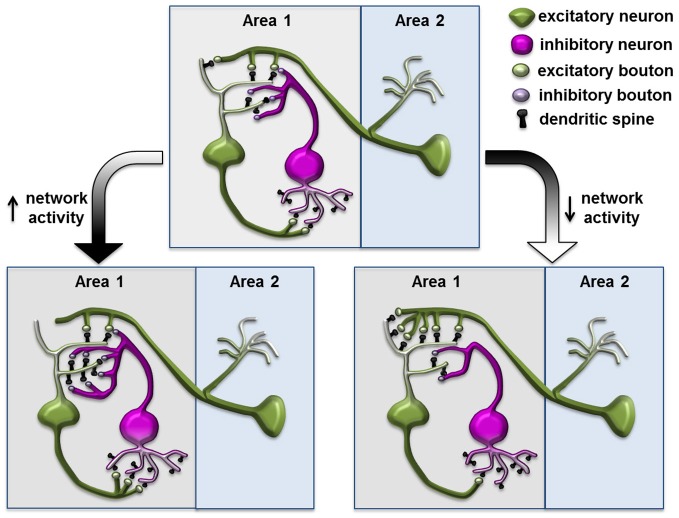
**Structural plasticity of inhibitory synapse in a neuronal network during homeostasis**. Increased network activity leads to reduced postsynaptic density on the excitatory neuron and increased synapses and spouting on the inhibitory neuron through connections to the excitatory neuron; conversely, decreased network activity leads to increased postsynaptic density on the excitatory neuron and reduced inhibitory elements on the inhibitory neuron. This diagram is based on the homeostatic network theory and is inspired by the schematics from Turrigiano and Nelson (2004); Butz et al. (2009b) and Gambino and Holtmaat (2012).

Many previous studies focused on modification of excitatory synapses formed on spines, partly due to their accessibility in morphological studies. Now, with improved methods to label and monitor inhibitory synapses *in vivo*, researchers have found a surprising degree of dynamics in inhibitory synapses and its potential role in regulating network activity within complex plastic events. In response to the chronic reduction or elevation of input activity, in addition to the homeostatic regulation of the principle neuron itself, the inhibitory neuron in the network can modify its connectivity to both the excitatory input and postsynaptic principle neurons, and contribute to counterbalancing the increased network activity (Figure 3; Turrigiano and Nelson, 2004). The inhibitory input within the network can, therefore, function as a perfect site for actions of homeostatic plasticity (Turrigiano and Nelson, 2004; Butz et al., 2009a; Turrigiano, 2012). This theory is strongly supported by previous experiments demonstrating functional homeostasis in a network (Hensch et al., 1998; Rutherford et al., 1998; Desai et al., 2002; Kilman et al., 2002), while emerging evidence also demonstrate that reorganization and changes in the dynamics of inhibitory synapses might contribute to network stabilization.

In the mouse barrel cortex, prolonged single whisker stimulation led to elevated neuronal activity and a transient increase of spine numbers in the corresponding cortical barrel. Subsequently, total synaptic density returned to pre-stimulation levels while only the inhibitory GABA synapses were maintained, potentially compensating for increased sensory input through elevated inhibitory input in the circuit (Knott et al., 2002). Two recent studies specifically labeled and studied cortical inhibitory interneurons in response to sensory deprivation. In the mouse visual cortex, neuropeptide Y (NPY)- positive inhibitory interneurons in layers 1 and 2/3 receive glutamatergic excitatory input on dendritic spines, while sending inhibitory signals to excitatory neurons through axonal boutons (Keck et al., 2011). Seventy-two hours after focal retinal lesions, the spine number in these inhibitory neurons was significantly reduced in the LPZ. In parallel, the number of their axonal boutons also decreased, suggesting a lower level of inhibition was induced by the loss of excitatory input in the network. Another study focused on layer 2/3 interneurons in the visual cortex, where binocular deprivation specifically increased retractions of the branch tips, while the monocular deprivation induced dynamic dendritic arbor rearrangements and reduced axonal bouton numbers onto layer 5 pyramidal apical dendrites (Chen et al., 2011). In both cases, the structural changes in inhibitory neurons could lead to reduction of overall inhibitory drive and serve as a part of the homeostatic response toward sensory deprivation.

The dendritic spines of cortical pyramidal neurons contain both excitatory synapses receiving excitatory input from intracortical axons and thalamocortical axons, as well as inhibitory synapses from local interneurons (Kubota et al., 2007; Gambino and Holtmaat, 2012). Using fluorescent-tagged gephyrin as a marker for inhibitory synapses, two groups studied inhibitory synapses dynamics in excitatory cortical layer 2/3 pyramidal neurons through long-term *in vivo* imaging (Chen et al., 2012; van Versendaal et al., 2012). Both studies made similar observations that a high fraction of gephrin-labeled synapses, ~30–40%, are localized on dendritic spines, and that they are highly dynamic. Interestingly, a short period of monocular deprivation (1–4 days) caused a significant increase in eliminating inhibitory synapses and a decrease in adding newly formed inhibitory synapses, especially those present on dendritic spines. This large-scale pruning of the inhibitory synapses could lead to increased excitability and constitute a homeostatic response of pyramidal neurons to compensate for the loss of excitatory input.

## Implications of structural homeostasis in physiological and pathological conditions of the brain

Proper establishment and maintenance of functional circuits rely on the abilities of neural networks to adjust their excitability based on the input they received. The deficits in compensatory structural reorganization during development or in adulthood have been implicated in a number of brain disorders. Clinical pathology studies link abnormality in dendrite morphology and neuronal homeostasis with several types of neurodevelopmental disorders and psychiatric diseases (Ramocki and Zoghbi, 2008; Toro et al., 2010; Arguello and Gogos, 2012). Mutations in genes regulating synaptogenesis and neuronal circuit formation have been associated with the increased risk of mental illnesses (Toro et al., 2010; Wondolowski and Dickman, 2013). Moreover, structural alterations in specific neural circuits are observed in patients with chronic stress and depression (Castrén and Hen, 2013). Although the causal relationship between anatomical changes and pathological conditions has not yet been established, it is likely that the deficits in structural plasticity and neuronal homeostasis contribute to neurological symptoms. Findings generated using new paradigms and model systems that we described above will undoubtedly update our views on the wiring dynamics of the nervous system and provide us clues to better understand these neurological disorders.

There are also emerging links between neuronal homeostasis and physiological functions of the brain. A particularly noteworthy area of research links sleep, an essential part of animal physiology, with synaptic homeostasis. Despite biological and clinical significance and decades of intense research, the function of sleep remains elusive. A hypothesis proposed by Tonini and Cirelli suggested that the main purpose of sleep is to produce global weakening of synaptic connections that were added or strengthened through experience and learning during the waking period (Tononi and Cirelli, 2003, 2006). The connection between sleep and synaptic homeostasis is supported by experiments carried out in *Drosophila*, where sleep was associated with widespread reduction in synapse number and level of molecules functioning as critical synaptic components (Bushey et al., 2011). In addition, there is evidence suggesting that the structural synaptic plasticity in the zebrafish circadian circuit is under both circadian and sleep-related homeostatic regulation. Sleep deprivation leads to increased synapse number along the axons projecting to the target tissue (Appelbaum et al., 2010). Although structural alterations in large areas of the brain occurring during sleep are yet to be confirmed in mammals, the concept of global synaptic downscaling during sleep is supported by recent findings in mammalian systems (Chauvette et al., 2012; Grosmark et al., 2012). This intriguing hypothesis indeed presents a possible explanation for the necessity of sleep in all animals, which is to ensure neuronal homeostasis and allow the Hebbian form of plasticity to occur daily throughout life.

## Conclusions

Studies we discussed here support structural homeostasis as an important component of neuronal plasticity and open up new areas for future investigations. At the same time, they add to the existing complexity of the array of mechanisms regulating neuronal form and function in the plastic events that lead to adaptation and learning. Alterations in morphology and connectivity are powerful ways to react to the sustained change in global activity, consolidate modifications in synaptic strength and efficacy, and strongly influence subsequent functional and behavioral adaptations. The capacity for structural homeostasis, therefore, could potentially serve as the target for regulatory mechanisms that shift the balance between wiring stability and flexibility within specific circuits and developmental stages.

Many questions remain unanswered in terms of the function and mechanism of structural homeostasis and its interactions with other forms of plasticity. For example, how do synapses, neurons or circuits sense the activity perturbation and initiate structural modification during homeostasis? What is the sequence of events and their temporal scale? Are there shared cellular and molecular pathways among structural and functional homeostasis, and Hebbian form plasticity? And how do they cooperate with development and functional adaptation? New experimental evidence obtained through updated technologies, such as simultaneous structural and functional imaging in behaving animals, as well as the reexamination of classic paradigms within the new context will both help us address these issues and improve our understanding of neuronal plasticity as a unity.

## Conflict of interest statement

The authors declare that the research was conducted in the absence of any commercial or financial relationships that could be construed as a potential conflict of interest.
